# Comparative genomics of *Paraburkholderia kururiensis* and its potential in bioremediation, biofertilization, and biocontrol of plant pathogens

**DOI:** 10.1002/mbo3.801

**Published:** 2019-02-27

**Authors:** Graciela M. Dias, Araceli de Sousa Pires, Vinicius S. Grilo, Michele R. Castro, Leonardo de Figueiredo Vilela, Bianca C. Neves

**Affiliations:** ^1^ Department of Biochemistry Chemistry Institute Federal University of Rio de Janeiro Rio de Janeiro Brazil; ^2^ Department of Biology Federal Institute of Rio de Janeiro Rio de Janeiro Brazil

**Keywords:** biocontrol of plant pathogens, biofertilization, bioremediation, comparative genomics, endophytic bacteria‐plant interaction, *Paraburkholderia kururiensis*

## Abstract

*Burkholderia* harbors versatile Gram‐negative species and is β‐Proteobacteria. Recently, it was proposed to split the genus in two main branches: one of animal and plant pathogens and another, *Paraburkholderia*, harboring environmental and plant‐beneficial species. Currently, *Paraburkholderia* comprises more than 70 species with ability to occupy very diverse environmental niches. Herein, we sequenced and analyzed the genome of *Paraburkholderia kururiensis* type strain KP23^T^, and compared to *P*. *kururiensis* M130, isolated in Brazil, and *P*. *kururiensis* susbp. *thiooxydans*, from Korea. This study focused on the gene content of the three genomes with special emphasis on their potential of plant‐association, biocontrol, and bioremediation. The comparative analyses revealed several genes related to plant benefits, including biosynthesis of IAA, ACC deaminase, multiple efflux pumps, dioxygenases, and degradation of aromatic compounds. Importantly, a range of genes for protein secretion systems (type III, IV, V, and VI) were characterized, potentially involved in *P*. *kururiensis* well documented ability to establish endophytic association with plants. These findings shed light onto bacteria‐plant interaction mechanisms at molecular level, adding novel information that supports their potential application in bioremediation, biofertilization, and biocontrol of plant pathogens. *P*. *kururiensis* emerges as a promising model to investigate adaptation mechanisms in different ecological niches.

## 1 INTRODUCTION

The genus *Burkholderia* harbors Gram‐negative, rod‐shaped, motile, non‐spore forming bacterial species, and belong to class β‐Proteobacteria. It comprises a versatile group of species, adapted to a wide diversity of niches, ranging from free‐living to plant‐ and animal‐associated organisms, some of them widely studied as human pathogens (e.g *Burkholderia cenocepacia* group) (Eberl & Vandamme, 2016). Several reports based on phylogenetic analysis have demonstrated that these members are divided in two groups: one containing mainly human, plant, and animal pathogens, while the second group consists of plant‐associated beneficial and environmental species (Estrada‐De Los Santos, Rojas‐Rojas, Tapia‐García, Vásquez‐Murrieta, & Hirsch, 2016; Estrada‐De Los Santos, Vinuesa, Martínez‐Aguilar, Hirsch, & Caballero‐Mellado, 2013; Gyaneshwar et al., 2011). Sawana and collaborators have proposed a reclassification of the second group, composed mainly of plant growth‐promoting bacteria (PGPB) as *Paraburkholderia*, based on new molecular markers (Sawana, Adeolu, & Gupta, 2014).

Besides promoting plant growth, PGPB species can also improve nutrient uptake, increased stress tolerance, induction of systemic resistance, and confer protection against plant pathogens (Segura, De Wit, & Preston, 2009; Vitorino & Bessa, 2017). Therefore, the novel genus has gained considerable importance owing to their promising potential in biocontrol of plant pathogens, biofertilization, bioremediation, and their low capacity of to infect humans (Angus et al., 2014; Estrada‐De Los Santos et al., 2016; Sawana et al., 2014).

The first *Paraburkholderia* species that gained attention due to their ability to promote benefits to plants were *P*. *vietnamiensis* (Trân Van, Berge, Ngô Kê, Balandreau, & Heulin, 2000) and *P*. *kururiensis*, the latter formerly named *B*. *brasilensis* (Baldani, Caruso, Baldani, Goi, & Dobereiner, 1997; Baldani, Oliveira et al., 1997) and *B*. *kururiensis* (Estrada‐De Los Santos, Bustillos‐Cristales, & Caballero‐Mellado, 2001; Mattos et al., 2008) The number of species described within the genus has increased rapidly with genome sequencing data, thus highlighting the importance of plant‐bacteria interaction of *Paraburkholderia* (Eberl & Vandamme, 2016; Suarez‐Moreno et al., 2010). For example, *P*. *phytofirmans* PsJN stimulates the plant growth and reduce plant disease severity caused by a virulent strain of *Pseudomonas syringae* pv tomato in *Arabidopsis* plants (Timmermann et al., 2017). Potential new species of *Paraburkholderia* were isolated from Brazilian Atlantic Forest (Mata Atlântica) and *Cerrado* soils. These species are characterized by their ability of nodulate and fix nitrogen (Bournaud et al., 2017; Dall'Agnol et al., 2016, 2017). The beneficial interaction between plant and bacteria is critical to agriculture and could improve soil fertility, crop yield, and therefore reduce the negative impacts of fertilizers on the environment. Important determinant factors to strong plant‐bacteria interaction are the ability to produce antimicrobial compounds, secondary metabolites, detoxification of reactive oxygen species (ROS), excreted siderophores, and effector proteins that modulate the host through protein secretion systems (Brader, Compant, Mitter, Trognitz, & Sessitsch, 2014).

Beneficial plant‐bacteria interactions have been studied for several years, and the knowledge about different interaction mechanisms has increased exponentially with the advent of next generation sequencing techniques. However, in the case of endophytic beneficial diazotrophic bacteria, such as *P*. *kururiensis*, very little is known about the molecular mechanisms involved in the plant‐bacteria cross‐talk. Thus, this work was focused at sequencing and annotating the genome of *P*. *kururiensis* KP23^T^, the type strain isolated from an aquifer polluted with trichloroethylene (TCE) in Japan, and comparing it to *P*. *kururiensis* M130, isolated from the roots of rice plants in Brazil, and *P*. *kururiensis* susbp. *thiooxydans*, isolated from rhizosphere soil of tobacco plant in Korea. Accordingly, phylogenetic analysis based on the 16S rRNA gene sequence and Multilocus Sequence Analysis (MLSA) demonstrated that these three strains are inserted into the environmental clade (Depoorter et al., 2016; Estrada‐De Los Santos et al., 2013, 2016). Characterizing these three *P*. *kururiensis* isolates from different sources and geographic locations by comparative genomic analyses revealed the presence of important genes in the process of colonization and adaptation in plants, such as flagella, multidrug efflux pumps, and dioxygenases. Importantly, a range of gene clusters for protein secretion systems (type III, IV, V, and VI) were observed and analyzed, some of which are potentially involved in *P*. *kururiensis* well documented ability to establish endophytic association with plants and its protective effect against pathogens. These findings shed light onto *P*. *kururiensis*‐plant interaction mechanisms at molecular level, and add unprecedented information to support the potential application of *P*. *kururiensis* in bioremediation, biocontrol of plant pathogens and biofertilization.

## 2 MATERIAL AND METHODS

### 2.1 Bacterial culture

Bacterial strain *P*. *kururiensis* strain KP23^T^ (LMG19447), originally isolated from a trichloroethylene‐polluted site in Japan (Zhang et al., 2000), was stored until use in Lysogeny Broth (LB; 10 g/L Tryptone, 5 g/L Yeast Extract, 5 g/L NaCl; Sigma‐Aldrich, St. Louis, USA) supplemented with 20% (v/v) glycerol, at −80°C. Components of storage, culture, and production media were obtained from Sigma‐Aldrich (St. Louis, USA). The strain was grown at 30°C in Lysogeny Broth for 24 hr, with agitation (200 rpm) for genomic DNA purification.

### 2.2 Genomic DNA preparation

The DNA genomic was extracted with the Blood and Cell Culture DNA Maxi Kit (Qiagen, Hilden, Germany), according to the manufacturer's instructions. DNA concentration was estimated by measuring the absorbance at 260 nm, samples with *A*_260_/*A*_280_ ratio of 1.7–2.0 were regarded as pure.

### 2.3 Genome sequencing of *P*. *kururiensis* KP23^T^

Genomic DNA was sequenced at Eurofins Genomics (Louisville, USA), using paired‐end sequencing on a MiSeq Illumina platform. The sequencing resulted in 2,979,894 reads with 100× coverage. The reads were assembled using SPAdes, with default k‐mer sizes (21, 33, 55, 77) (Bankevich et al., 2012). The quality control of the sequence was performed with QUAST (Gurevich, Saveliev, Vyahhi, & Tesler, 2013). The subsequent assembled produced in 153 contigs.

### 2.4 Annotation and comparative genomics

The three genomes were annotated by PROKKA (Seemann, 2014). Proteins were identified by domains for specific functions by InterProScan (Jones et al., 2014), by clusters of orthologous groups (COG) (Tatusov, Galperin, Natale, & Koonin, 2000), and KEGG database (http://www.genome.jp/kegg/pathway.html). For comparative genomics, GET_HOMOLOGUES was used (Contreras‐Moreira & Vinuesa, 2013). The protein secretion systems were predicted with a combination of two tools, MacSysFinder (Abby, Néron, Ménager, Touchon, & Rocha, 2014) and T346 hunter (Martínez‐García, Ramos, & Rodríguez‐Palenzuela, 2015). We computed T6SS clusters in the genomes of *P*. *kururiensis* strains based on COG categories, as described by Boyer and collaborators (Boyer, Fichant, Berthod, Vandenbrouck, & Attree, 2009). Along with the genome sequence of type strain KP23^T^, obtained in this study, two *P. kururiensis* genomes were used for comparative analyses: *P*. *kururiensis* M130 (PRJNA183622), isolated from the roots of rice plants in Brazil (Coutinho, Mitter et al., 2013; Coutinho, Passos et al., 2013) and *P*. *kururiensis* subsp. *thiooxydans* ATSB13^T^ (NBRC107107) (PRJDB254) (Anandham et al., 2009), both available in the NCBI genome database.

## 3 RESULTS AND DISCUSSION

### 3.1 General features of *Paraburkholderia kururiensis* genomes

**Figure 1:**
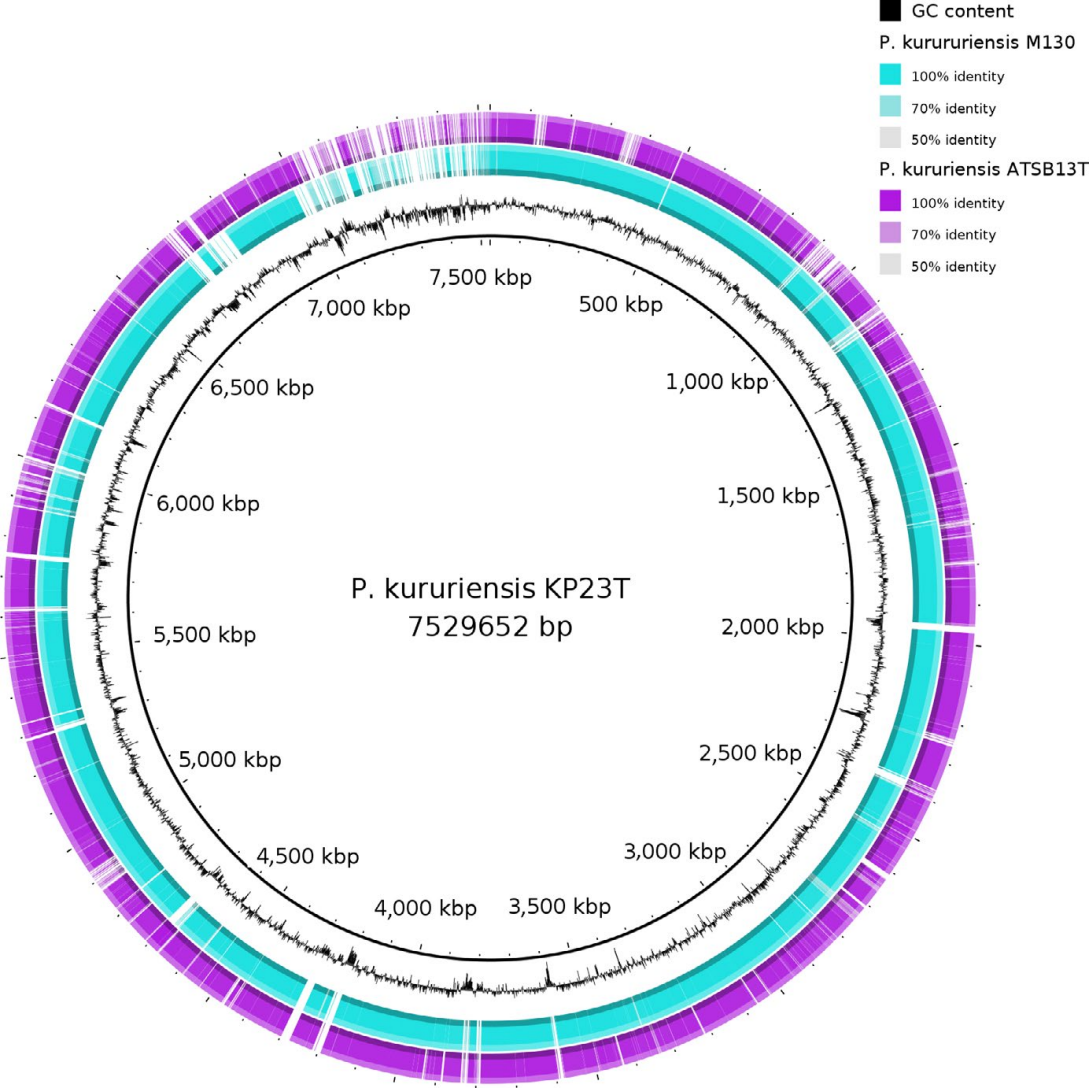
Genome atlas of *Paraburkholderia kururiensis* strains. The figure is based on blastn analyses of *P. kururiensis* KP23^T^ against M130 and ATSB13^T^ genomes. The innermost circle shows GC content. The cyan circle indicates the genome of *P. kururiensis* M130 and the purple circle shows *P. kururiensis* ATSB13^T^ genome. The white gaps indicate the absent sequences in the M130 and ATSB13^T^ genomes

The genome of *P*. *kururiensis* KP23^T^ consists of 7,529,652 bp with a GC content of 64.8%. A total of 6,596 CDS was identified in this genome, from which 2,367 (35%) are hypothetical proteins. To perform the comparative analysis, we re‐annotated two different *P*. *kururiensis* strains, M130 and ATSB13^T^ already available in the NCBI database, with genomes sizes of 7.1 and 6.7 Mb, and GC content of 65.03%, 63.87% respectively (Table 1). In order to identify the total number of orthologs shared among all strains, we combined three algorithms: COGtriangles, OrthoMCl, and BDBH. *P*. *kururiensis* core was composed of 4,711 ortholog genes, from which 1,378 (29%) were annotated as hypothetical proteins. COG database was used to categorize the proteins of the three genomes of *P*. *kururiensis* isolates. The COG categories demonstrated a highly similar distribution for the three genomes. The percentage of genes in the most abundant categories were for energy production and conversion (C), amino acid transport and metabolism (E), and carbohydrate transport and metabolism (G) suggesting that these three strains present a similar lifestyle. In addition, we performed a blastn atlas of the genomes using BRIG tool, which demonstrates the gene conservation among the *P*. *kururiensis* strains (Figure 1). Further analyses of these strain‐specific CDS may provide clues to the phenotype and the specific environmental adaptations of each strain.

**Table 1:** General features of *P. kururiensis* genomes

Genomes/features	KP23^T^	M130	ATSB13^T^
Size	7,529,652	7,128,857	6,795,583
GC content (%)	63,8	65,03	63,87
CDS	6,596	6,266	6,086
Hypothetical proteins	2,367	2,117	2,159
tRNAs	58	59	60
rRNAs	2	2	2
Sample origin	TCE‐polluted Aquifer	Rice plant	Tobacco plant

### 3.2 Comparative genomic analysis

#### 3.2.1 Plant growth‐associated gene factors

Many endophytic bacteria confer several benefits to the host plant, including growth promotion and protection from pathogens. A commonly associated mechanism involve the nitrogen fixation and the production of indole‐*3*‐acetic acid (IAA), that belongs to the auxin phytohormone family, and 1‐aminocyclopropane‐1‐carboxylate (ACC) deaminase, that controls the amount of ethylene levels in plants (Olanrewaju, Glick, & Babalola, 2017; Santoyo, Moreno‐Hagelsieb, del Carmen Orozco‐Mosqueda, & Glick, 2016). In the present work we identified the genes involved in the nitrogen fixation and production of these two hormones in all three strains.

The three strains present the genes involved to nitrogen fixation (*nifHDK*) and nodulation. The genes are annotated as nitrogenase iron protein, nitrogenase molybdenum‐iron protein alpha chain, and nitrogenase molybdenum‐iron protein beta chain, respectively. In the adjacently region, we found the genes *nifNE*, responsible by the biosynthesis of the iron‐molybdenum cofactor (FeMo‐co). In addition, we found the genes *nifABZWT* that are located next to the cluster *nifNE‐nifHDK*. These genes are also associated to nitrogenase complex biosynthesis (Sickerman, Rettberg, Lee, Hu, & Ribbe, 2017). The ability of these strains and other strains of genus *Paraburkholderia* to fix N_2_ has been demonstrated in previous reports through several analyses such as acetylene reduction activity (ARA) and presence/absence of *nifH*. (Estrada‐De Los Santos et al., 2001; Martínez‐Aguilar, Díaz, Peña‐Cabriales, & Caballero‐Mellado, 2008; Mattos et al., 2008). As shown in Table 2, the overall gene counting for nitrogen fixation is similar in the three strains: 20 genes in strain M130 and 19 in stains KP23^T^ and ATSB13^T^.

**Table 2:** Predicted genes involved in bacteria‐plant interaction

Function	*P. kururiensis* genomes		
	KP23^T^	M130	ATSB13^T^
Plant growth			
ACC deaminase	1	1	1
Pyrroloquinoline quinone (*pqqBCDE*)	4	4	4
IAA biosynthesis (triptofan‐2‐monoxigenase)	1	1	1
2‐Phenylethanol	2	2	2
Nitrogen fixation	19	20	19
Plant polymer degradation/modification			
Cupin domains	35	35	35
Alpha/beta hydrolase	25	28	29
Glycosyl hydrolase	12	10	9
Motility and chemotaxis			
Tad system	8	8	8
Type III secretion system flagellar	58	57	59
Methyl accpeting chemotaxis	40	40	31
Detoxification			
Glutathione S‐transferase	22	23	21
Nitrilase	4	4	3
Superoxide dismutase	3	3	3
Hydrogen cyanide	15	12	12
Catalase/peroxidase	3	3	4
Short‐chain‐enoyl‐CoA hydratase	6	6	6
Quercertin 2,3 dioxygenase	3	3	3
Mannitol dehydrogenase	3	3	3
2‐dehydropantoate 2‐reductase	2	2	3
Multidrug efflux			
Major facilitator superfamily	102	107	97
AcrB/AcrD/AcrF family	16	16	16
RND efflux pump	17	22	20
Secretion and delivery system			
Type II secretion system	20	22	22
Type III secretion system	9	9	9
Type IV secretion system	6	‐	6
Type V secretion system	15	8	7
Type VI secretion system	41	41	29
The numbers indicate the gene copies; ‐ absence of genes			

Our findings also corroborate with new *Paraburkholderia* genomes recently published, such as *P*. *caballeronis* Tne‐841, *P*. *tropica* Ppe8, and *P*. *phymatum* STM815^T^ (Moulin, Klonowska, & Riley, 2014; Silva et al., 2017, Rojas‐Rojas et al., 2017), which have the ability to fix nitrogen and establish endophytic association. According to previous studies (Baldani, Caruso et al., 1997; Mattos et al., 2008), *P*. *kururiensis* lacks the ability of nodulation, although we identified three copies of *nodD* genes in all strains. These genes present high similarity (>60%) to *nodD* genes of nodulating *Paraburkholderia* species, including *P*. *nodosa*,* P*. *phenazinium*,* P*. *bannensis,* and *P*. *tropica*. Interestingly, *nodD* genes have been previously associated with LPS modifications in the interface between *Rhizobium* and host plants (Brencic & Winans, 2005).

We found one potential biosynthesis pathway for IAA and indole‐3‐acetamide (IAM). In this pathway, tryptophan released by the roots is converted to IAM by tryptophan‐2‐monooxigenase and then to IAA by amidase, while to ethylene biosynthesis, all the strains encode 1‐aminocyclopropane‐1‐carboxylate (ACC) deaminase, which cleaves ACC, the immediate precursor of ethylene in plants, decreasing the ethylene levels in plants (Glick, 2015).

A model has been previously proposed for a fine modulation between these phytohormones. The IAA production by plants and bacteria activate the transcription of the plant enzyme ACC synthase, leading to an increase of ethylene levels, which may cause damage to plants. However, the ACC released by the plants can be taken up by endophytic bacteria, decreasing the ethylene levels and promoting plant growth (Gamalero & Glick, 2015; Glick, 2014).

Evidence that these phytohormones produced by bacteria can cause plant growth promotion has been demonstrated in some species of genus *Burkholderia* and *Paraburkholderia*, such as *P*. *phytofirmans* and *P*. *kururiensis* (Castanheira et al., 2016; Mattos et al., 2008). In fact, some studies have demonstrated the ability of plant growth promoting bacteria (PGPB) to produce phytohormones, which makes them attractive for use in agriculture, as an alternative to chemical fertilizers, pesticides and herbicides (Babalola, 2010; Glick, 2014).

Other important benefit from PGPB is the protection against pathogens by producing several substances with antimicrobial activity. In *P*. *kururiensis,* we found a large repertoire of genes involved in protection, such as genes involved to putrescine biosynthesis and 4‐hydroxybenzoate (*ubiC)*. Likewise, production of 4‐hydroxybenzoate has been described in several PGPB genomes, including *Pseudomonas fluorescens* Pf‐5, *Pseudomodas putida* UW4, *Enterobacter sp*. 638, and *Mesorhizobium amorphae* (Duan, Jiang, Cheng, Heikkila, & Glick, 2013; Hao et al., 2012; Paulsen et al., 2005; Taghavi et al., 2010). This product is synthetized by the enzyme UbiC from chrorismate degradation. We also found two putative genes responsible to convert the phenyl‐acetaldehyde into 2‐Phenylethanol, showing a high similarity (>70%) with genes from PGPB *Enterobacter* sp. 38 (Taghavi et al., 2010). This compound also is found in other bacterial and yeast species (Liu, Chen, Li, Yang, & Long, 2016; Tasin et al., 2018). Given this reports, we suggest that the production of these compounds by *P*. *kururiensis* may account for its ability to control fungal and bacterial plant pathogens.

#### 3.2.2 Plant polymer hydrolases and protective secondary metabolites

Gene products involved in plant cell wall degradation are essential to successful colonization and spread of endophytic bacteria inside plants, and many belong to the glycoside hydrolase family. We found a high number of genes for glycosyl hydrolases, alpha/beta hydrolases and cupin domains (Table 2). These genes have also been found in *Burkholderia* spp., *Enterobacter* sp. and *Bacillus mycoides*, the latter was isolated from potato endosphere, and demonstrated the upregulation of several alpha‐hydrolases in response to potato root exudates (Ali, Duan, Charles, & Glick, 2014; Taghavi et al., 2010; Yi, de Jong, Frenzel, & Kuipers, 2017). Thus, these genes seem essential for endophytic bacteria, revealing the importance of these enzymes in this colonization process. On the other hand, proteins harboring cupin domains are mostly annotated as monoxygenases and dioxygenases that play a key role in degrading aromatic rings in aromatic metabolism. Metagenomic analyses of biodiversity of Cerrado, a savannah‐like biome in the Midwestern region of Brazil, revealed the presence of new genes encoding dioxygenases, which could play a role on the resistance phenotype and degradation of aromatic rings (dos Santos, Istvan, Noronha, Quirino, & Krüger, 2015).

Additionally, we also found the biosynthetic gene clusters for aryl‐polyenes (APEs) in all three *P*. *kururiensis* genomes. Aryl‐polyenes (APEs) are pigments similar to carotenoids and have been associated to bacterial protection against light‐induced damage and reactive oxygen species (ROS) (Cimermancic et al., 2014; Schoener et al., 2016). In all three genomes, the clusters comprise 40 kb and harbor approximately 45 genes, from which 35% of the genes show similarity with APE cluster from *V*. *fischeri*. Therefore, the presence of this system may represent an additional weapon against the stress‐prone oxidative environment within the plant tissues.

#### 3.2.3 Motility and chemotaxis

Successful establishment on plant roots and other plant parts by PGPB bacteria involves many processes, including motility and chemotaxis. These colonization processes are well known in the host‐pathogen interaction (Segura et al., 2009).

The genomes of *P*. *kururiensis* revealed genes related to chemotaxis, flagella biosynthesis and pili biosynthesis, including 10 genes encoding two‐component systems (CheA‐CheYBB), 40 genes for methyl‐accepting chemotaxis (MCP) in KP23^T^ and M130 and 31 to ATSB13^T^ (Table 2). We identified the presence of 38 genes associated with flagella biosynthesis, including the *flg* and *fli* operons. Previous studies demonstrated that, in the endophyte *Azoarcus* sp., deletion of genes involved in motility prevented twitching and motility as well as the endophytic root colonization of rice plants (Böhm, Hurek, & Reinhold‐Hurek, 2007). In addition, transcriptomic analysis of strain M130 demonstrated that genes related to bacterial motility, chemotaxis and adhesion were induced in the presence of rice plants extract (Coutinho, Licastro, Mendonça‐Previato, Cámara, & Venturi, 2015).

In the three genomes, we also identified three tight adherence (*tad*) systems. These systems have been reported as adherence, attachment, and colonization factors in a wide variety of bacteria (Motherway et al., 2011; Nykyri et al., 2013; Pu & Rowe‐magnus, 2018). Their role was initially assessed in silico and experimentally confirmed in some PGPB species, such as *Pseudomonas chlororaphis* and *Mesorhizobium amorphae* (Chen et al., 2015; Haq, Graupner, Nazir, & van Elsas, 2014; Shen, Hu, Peng, Wang, & Zhang, 2013). Therefore, the presence of motility, chemotaxis, and colonization‐associated genes in *P*. *kururiensis* strongly suggests a possible advantage in plant colonization.

#### 3.2.4 Detoxification and plant defense mechanisms

The colonization process of bacteria in plants can produce large amounts of ROS, causing negative effects to bacteria. Therefore, the detoxification process is an important feature to protect the bacteria under oxidative stress in the plant colonization (Wani, Ashraf, Mohiuddin, & Riyaz‐Ul‐Hassan, 2015). In plant‐associated bacteria, several genes have been described as responsible for protecting the bacteria under stress, during the colonization process in host plants. We found a versatile repertoire of genes for detoxification in the three *P*. *kururiensis* strains, including genes for glutathione S‐transferase, hydrogen cyanide synthase, superoxide dismutase, catalase, mannitol dehydrogenase, and peroxidase (Table 2). Accordingly, comparative genomic analyses revealed the presence of stress‐associated genes in several genomes of endophytic bacteria (Ali et al., 2014).

In addition, we also found a set of enzymes, such as nitrilases, dehydrogenases, hydratases, and dioxygenases. For example, all three genomes of *P*. *kururiensis* encode quercetin 2,3‐dioxygenase, an enzyme that converts quercetin in 2‐protocatechuoyl‐phloroglucinol carboxylic acid and carbonoxide. The quercetin is a flavonoid with antibacterial activity, a primary plant defense agent, which inhibits bacterial DNA gyrase and induces DNA cleavage (Plaper et al., 2003). This enzyme has also been found in the soil bacterium *B*. *subtilis* and some fungi species (Fusetti et al., 2002; Hirooka & Fujita, 2010). Therefore, the presence of quercetin 2,3‐dioxygenase enables bacteria to avoid this host plant defense mechanism. Nitrilases are enzymes that catalyze the hydrolysis of nitrile compounds to the corresponding carboxylic acids and ammonia, and can be applied to the synthesis of industrial carboxylic acids and bioremediation of cyanide and toxic nitriles (Howden & Preston, 2009; Segura et al., 2009). We found the cluster *hcnCAB* in the three genomes of *P*. *kururiensis*, composed by three genes, *hcnA, hcnB,* and *hcnC*, which encode the subunits A, B, and C, respectively, of the enzyme HCN synthase. This product inhibits cytochrome c oxidase as well as other important metalloenzymes (Nandi, Selin, Brawerman, Fernando, & de Kievit, 2017). In fact, hydrogen cyanide (HCN) is a secondary metabolite produced by several bacteria. It has also been described in several PGPB acting as a biocontrol of plant pathogens.

#### 3.2.5 Multidrug efflux pumps

The efflux pumps are versatile structures capable of extruding toxic compounds, such as antibiotics, heavy metals and solvents. These systems are usually encoded in the chromosome and are highly conserved in bacteria (Alvarez‐Ortega, Olivares, & Martinez, 2013; Martínez et al., 2009). We have found a total of 155 genes involved in multidrug resistance (MDR) efflux pumps in the three genomes of *P*. *kururiensis*. This high number of MDR corroborates with previous reports which showed that MDR pumps are widely present in soil and/or plant‐associated bacteria (Konstantinidis & Tiedje, 2004). All efflux transporters identified in the three genomes belong to Resistance Nodulation cell Division (RND) efflux and Major superfamily (MFS)‐type, such as AcrAB, MdtABC, and EmrAB. Several authors have demonstrated that efflux pumps of endophytic *P*. *putida*,* E. chrysanthemi* and *S. maltophilia* extend beyond antibiotic resistance, they are important for first steps of colonization and survival in plant tissues (Espinosa‐Urgel, Salido, & Ramos, 2000; Mukherjee & Roy, 2016). In addition, they can also be involved in biofilm formation, quorum sensing, chemotaxis, and motility (Alvarez‐Ortega et al., 2013; Espinosa‐Urgel & Marqués, 2016; Martínez et al., 2009).

#### 3.2.6 Degradation of aromatic compounds

Aromatic compounds are the most prevalent organic pollutants in the environment and could be extremely toxic to humans, increasing the risk of causing cancer and other cellular modifications. Degradation of these compounds are dominated by bacteria and fungi, as these substances can be used as source of carbon and energy, having a central role in the recycling of carbon (Cao, Nagarajan, & Loh, 2009; Fuchs, Boll, & Heider, 2011; Seo, Keum, & Li, 2009).

Putative genes coding for the degradation of several aromatic compounds were identified in all three strains, such as phenol, benzene, benzoate, toluene, 3‐phenylpropionic acid, and phenylacetate degradation (Table 2 and Appendix Table A1). Primarily, the presence of these pathways may be directly involved in degradation of aromatic compounds originated within host plants (e.g. lignin). These findings corroborate previous reports that demonstrate the ability of *P*. *kururiensis* KP23^T^ and M130 to grow with phenol*,* toluene, and benzene (except M130), while some other closely related species were able to grow only with benzene (Caballero‐Mellado, Onofre‐Lemus, Estrada‐De Los Santos, & Martínez‐Aguilar, 2007; Gonzalez et al., 2013). In addition, Gonzalez and collaborators demonstrated that strain KP23^T^ in association with *Brassica napus* was able to tolerate and degrade much higher concentrations of different phenolic compounds, when compared to rhizobacterium *Agrobacterium rhizogenes* (Gonzalez et al., 2013). Several bacterial species that harbor these degradation pathways have been described as potential bioremediation agents, including *Stenotrophomonas* sp., *Pseudomonas fluorescens* Pf‐5 (Monisha, Ismailsab, Masarbo, Nayak, & Karegoudar, 2018; Mukherjee & Roy, 2016; Paulsen et al., 2005). Therefore, the presence of these pathways in *P*. *kururiensis* makes it a strong candidate for use in bioremediation of chemically impacted environments, with or without plant association.

#### 3.2.7 Quorum sensing and quorum quenching

The BraI/R is a classical quorum sensing system (QS) described in *P*. *kururiensis* and other *Paraburkholderia* species (Suárez‐Moreno, Caballero‐Mellado, & Venturi, 2008). The system employs N‐acyl homoserine lactone (AHL) signals and consists of two main genes (*braI* and *braR*) transcribed in the same operon, and the *rsaL* repressor, located between the other two genes (Choudhary et al., 2013). Other QS genes, the *xenI2*/*xenR2* system, are found in some PGPB species (e.g., *P*. *xenovorans*), but not in *P*. *kururiensis* strains. The BraI/R QS has been described as an important exopolysaccharide (EPS) regulator in several PGBP species (e.g. *P*. *kururiensis, P*. *unamae, and P*. *xenovorans)*. However, the phenotypes regulated by BraI/R are not completely understood, suggesting the presence of other regulatory systems (Coutinho, Mitter et al., 2013; Suarez‐Moreno et al., 2010). Previous work also revealed that the endophytic bacterium *P*. *kururiensis* M130 in the presence of rice plant macerate presents upregulation of BraI/R QS system (Coutinho et al., 2015).

Further comparative analysis showed the presence of other types of signaling systems in *P*. *kururiensis*, such as the diffusible signal factor (DSF). The DSF is produced by the RpfF enzyme of plant pathogen *Xanthomonas campestris* and has also been found in the *B*. *cenocepacia* complex. Blast analyses demonstrated that homolog *rpfF* of *P*. *kururiensis* had 77% identity to that of *B. cenocepacia* complex. This system has been associated with regulation of swarming motility, biofilm formation, and virulence in *B. cenocepacia* (Deng et al., 2012). Besides, this system has been also found in other members of genus *Burkholderia* and *Paraburkholderia* that confer benefits to their hosts (Suppiger, Aguilar, & Eberl, 2016).

Another important finding in *P*. *kururiensis* was the presence of lactonases (*aiiA* homologs), an enzyme responsible to degrade AHL molecules, thus inhibiting the production of QS signal molecules. This process is also known as quorum quenching (QQ). Enzymes involved in QQ have been described in a wide variety bacteria (see review by Kalia, 2013). Previous reports suggest that the co‐existence of QS and QQ can result in attenuation of pathogens, playing a potential role in biocontrol (Chan et al., 2011; Glick, 2015; Liu et al., 2017; Polkade, Mantri, Patwekar, & Jangid, 2016). Therefore, we believe that in *P*. *kururiensis* these two systems, QS and QQ, could play a pivotal role in establishing beneficial plant/bacteria associations and protection against plant pathogens.

#### 3.2.8 Type III secretion system

The Type III secretion system (T3SS) is components of two machineries: the flagellum, which is related to cell motility and non‐flagellar T3SS (NF‐T3SS), that is involved in the delivery of protein effectors into eukaryotic cells (Diepold & Armitage, 2015). The NF‐T3SS and flagellum consist of nine ubiquitous core proteins encoding a structural apparatus and accessory proteins that vary greatly in their number among the two systems (Abby & Rocha, 2012; Abrusci, McDowell, Lea, & Johnson, 2014).

**Figure 2:**
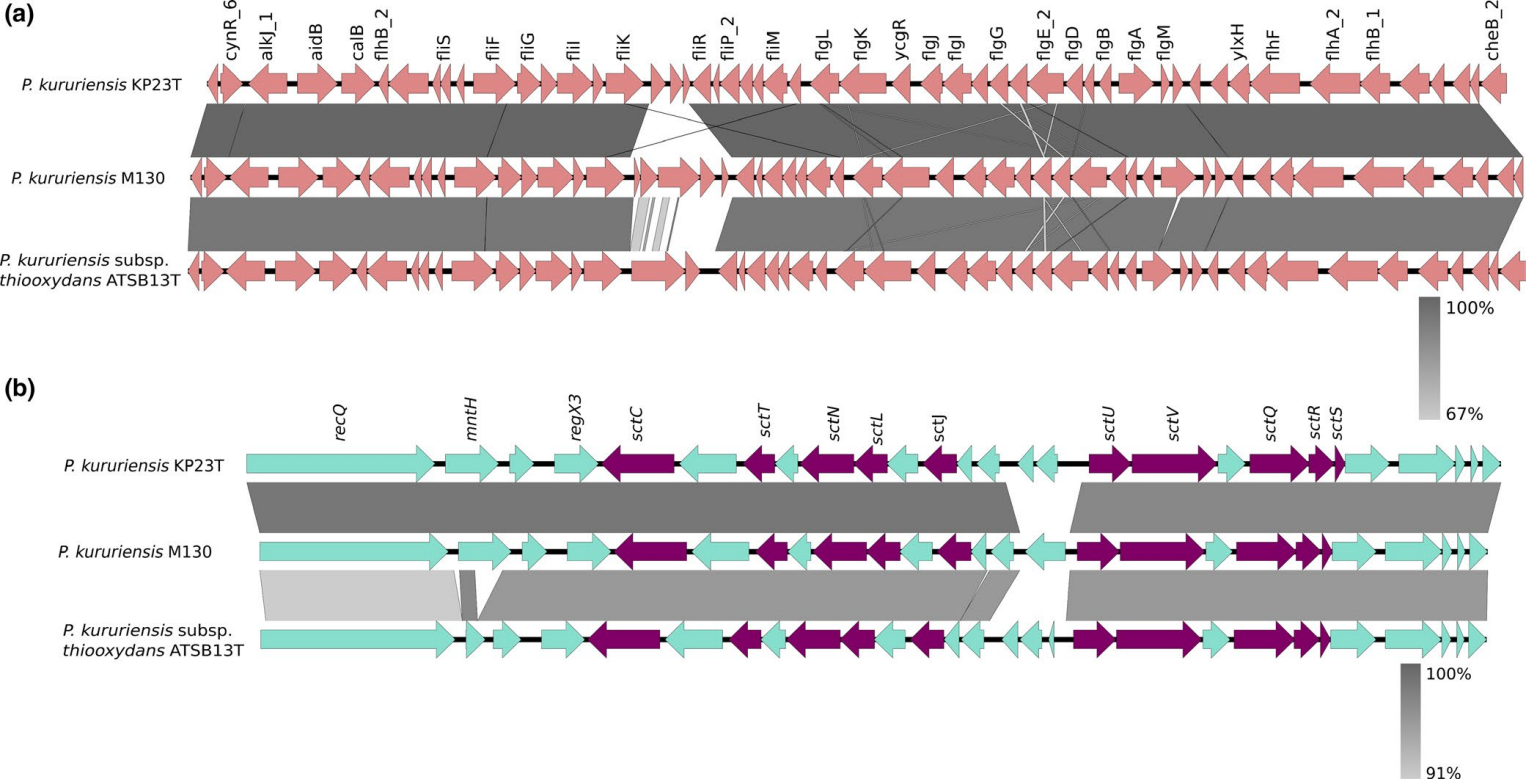
Gene organization of Type 3 secretion systems in *P. kururiensis*. The genes are represented by arrows that indicate direction of transcription. (a) Flagellar genes in the three strains. (b) NF‐T3SS in the three strains. The purple arrows indicate the conserved genes of NF‐T3SS and cyan arrows indicate the neighbor genes. The gray scale indicates the percentage of identity between the genomes. Gene prediction was performed by T346Hunter

**Figure A1:**
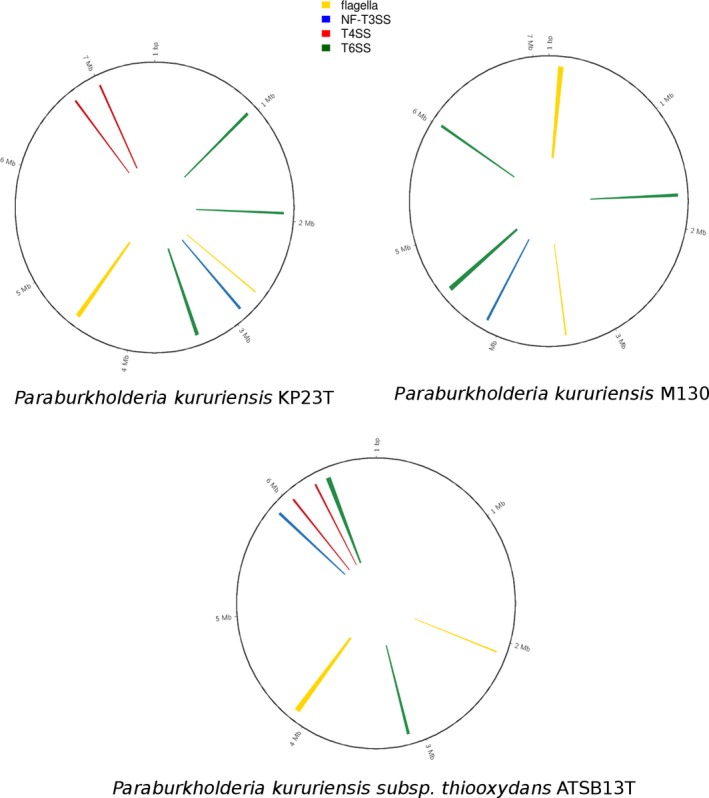
Predicted protein secretion systems in *P. kururiensis* strains. The color lines on the circles show the protein secretion systems localization in the genomes. Yellow lines represent the T3SS flagellar; blue lines represent the NF‐T3SS; red lines represent the T4SS; and green lines represent the T6SS. The protein secretion systems were predicted by T346Hunter.

The flagellar and NF‐T3SS clusters were found in all *P*. *kururiensis* strains (Figure 2, Appendix Figure A1 and Appendix Table A2). Blastn analysis revealed that this region shows high similarity (>75 and 80% coverage) to other members of genus *Paraburkholderia*, including *P*. *terricola*,* P*. *phenoliruptrix* BR3459a, and *P*. *graminis* PHS1. The three *P*. *kururiensis* strains harbor the *sctCTNLJUVQRS* genes. Interestingly, the presence of flagellum genes in the KP23^T^ contrasts with previous reports that this strain is non‐motile (Anandham et al., 2009; Zhang et al., 2000), suggesting there may be unidentified genetic and environmental factors involved in their expression.

The NF‐T3SS has been initially described in pathogens and phytopathogens. However, over recent years the number of endophytic strains that present the T3SS has increased. For example, experimental results of *P*. *fluorescens* Pf29Arp showed differences in expression levels of T3SS genes between wheat roots and necrotized roots by pathogenic fungus *Gaeumannomyces graminis* var. *tritici* (Ggt). Besides, T3SS mutants of *Bradyrhizobium s P*. SUTN9‐2 presented a decreased ability to colonize rice tissues (Marchi et al., 2013; Piromyou et al., 2015). In *P*. *kururiensis*, the core of NF‐T3SS (*SctSRQUJNT*) was shared between several endophytic bacteria, such as *B. rhizoxinica* HKI454, *B. terrae* BS001, *B. glumae,* and *B. phenoliruptrix* BR3459 (Haq et al., 2014). Taken together, these reports suggest that NF‐T3SS are essential to colonization and bacterial adaptation to different plant environments, mediating either pathogenic or beneficial associations.

#### 3.2.9 Type IV and V secretion system

The type IV secretion system (T4SS) is a highly versatile apparatus, commonly associated to DNA conjugative transfer, uptake, transformation and translocation (Juhas, Crook, & Hood, 2008). We found two clusters of T4SS (T4SS‐1 and T4SS‐2) in strains KP23^T^ and ATSB13^T^ (Appendix Figure A1 and Appendix Table A2). The T4SS‐1 contains genes involved in plasmid maintenance (*sopAB*) and plasmid transfer by conjugation, such as *tra* and *trb* genes, strongly indicating the presence of a conjugative plasmid. Blast analysis revealed high similarity to several genes of plasmid‐containing *Burkholderia* and *Paraburkholderia*, thus indicating the presence of a plasmid.

The second T4SS‐2 gene cluster (Appendix Table A2) contains *virB* homologs (43%), originally described in the plant pathogen *Agrobacterium tumesfascens* (Vergunst et al., 2000). This Type IV cluster is absent in the *B. pseudomallei* group, but is present in environmental strains, including *P*. *phymatum* PsJn and *P*. *terrae* (Haq et al., 2014). The role of Type IV secretion system in the plant‐bacteria interaction is not well understood, although some studies have demonstrated that it may also be involved in microbial competition (Angus et al., 2014; Souza et al., 2015).

The Type V secretion system (T5SS) is divided in five types T5aSS, T5bSS, T5cSS, T5dSS, and T5eSS (Fan, Chauhan, Udatha, Leo, & Linke, 2016). Four of them, T5aSS, T5cSS, T5dSS, and T5eSS, are characterized by the presence of translocator and passenger domains encoded in a single gene, known as the autotransporters. Type T5bSS, also known as two‐partner secretion (TPS) system, is encoded by two genes, one for the translocator and the other for passenger domain (Abby et al., 2016; Fan et al., 2016).

Overall, we identified 30 clusters belonging to three T5SS types (T5aSS, T5bSS, and T5sSS) in the three *P*. *kururiensis* genomes (Appendix Table A2). The number of clusters was variable between the three genomes: for T5aSS, we identified two clusters in strains KP23^T^ and M130; for T5bSS, eight clusters in KP23 and only three in M130 and ATSB13^T^; for T5cSS, the number in each genome varied from three, in strain M130, four in ATSB13^T^ and five in strain KP23^T^. For T5aSS, the mandatory domains are PF03797 (Autotransporter beta‐domain) and PF12951 (Passenger‐associated‐transport‐repeat); T5cSS presents PF03895 (Coiled stalk of trimeric autotransporter adhesin); T5bSS consists of PF03865 (Hemolysin secretion/activation protein ShlB/FhaC/HecB), PF17287 (POTRA domain), and PF08479 (POTRA domain, ShlB‐type); and finally the T5cSS type presents the PF03895 domain (Coiled stalk of trimeric autotransporter adhesin) (Appendix Table A3). The role of T5SS in the beneficial plant‐bacteria interaction has not yet been described in the literature and needs further investigation. However, we identified a large number of T5bSS clusters in KP23^T^ genome. Recent studies showed that T5bSS mediates the interbacterial competition or cooperation (Guérin, Bigot, Schneider, Buchanan, & Jacob‐Dubuisson, 2017). For example, in the case of *B. thailandensis* the TPS system confers the ability to antagonize competitors and promote the communication and cooperation between bacteria (Garcia, Anderson, Hagar, & Cotter, 2013; Garcia, Perault, Marlatt, & Cotter, 2016). This abundance of these clusters, especially in strain KP23^T^, suggests they may be important in the plant colonization process and/or interaction with the resident microbiome.

#### 3.2.10 Type VI secretion system

**Figure 3:**
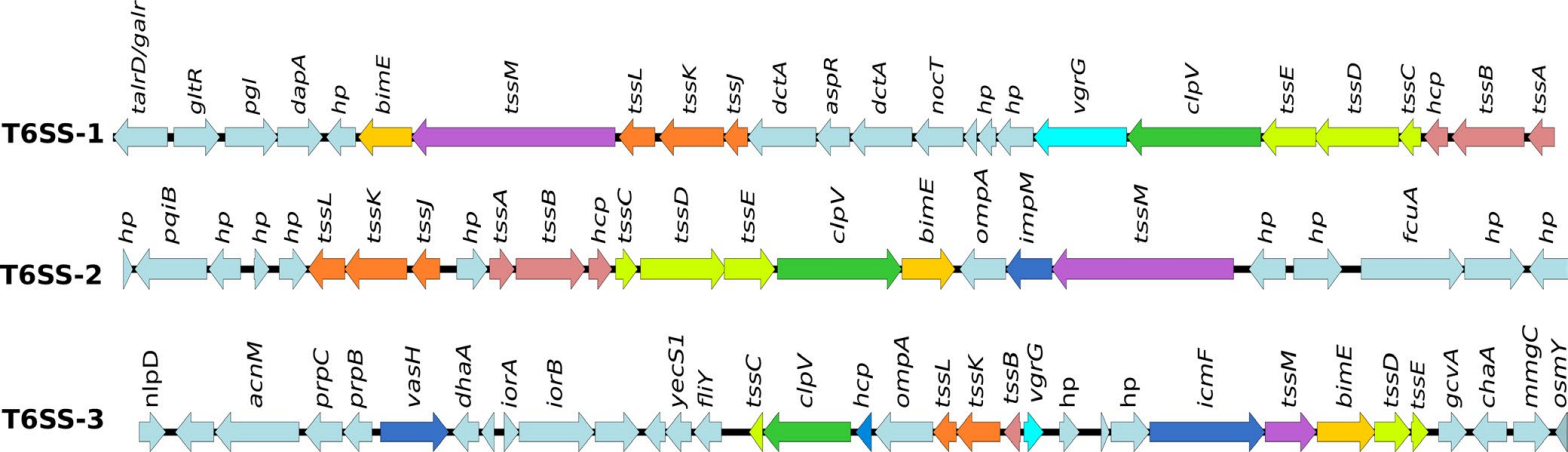
Gene organization of Type 6 secretion systems in *P. kururiensis*. The figure shows the three gene clusters coding for T6SS‐I, T6SS‐II and T6SS‐III. The genes are indicated by arrows and the direction of arrows represent the direction of transcription. The colored arrows indicate the conserved genes of T6SS, except the light blue that indicate the neighbor genes. The gray scale indicates the percentage of identity between the genomes. Gene prediction was performed by T346Hunter

The type VI secretion system (T6SS) was discovered in 2006 (Pukatzki et al., 2006). It has since been mainly associated with human pathogens, contributing to virulence in hosts. However, more recent studies have demonstrated that this system may play an important role in mediating interbacterial competition, biocontrol, or even contribute to virulence in plant‐pathogenic bacteria (Bernal, Allsopp, Filloux, & Llamas, 2017; Bernal, Llamas, & Filloux, 2018). Analysis of *P*. *kururiensis* genomes revealed the presence of three clusters of T6SS in strains KP23^T^ and M130 (T6SS‐1, T6SS‐2, T6SS‐3), but only two clusters (T6SS‐2 and T6SS‐3) in strain ATSB13^T^. The T6SS‐1, T6SS‐2, and T6SS‐3 clusters contain 33, 27, and 35 genes with a size of 30, 19, and 22 Kb, respectively (Figure 3, Appendix Figure A1 and Appendix Table A2). Of these, approximately 14 are included in the COG categories and classified as conserved T6SS genes. The clusters contain all genes required for a functional T6SS apparatus, including homologs of *tssM* and *tssL* (COG3523 and COG3455), ClpV (COG0542), *tssA* and *tssB* (COG3516 and COG3517), *tssC*,* tssD,* and *tssE* (COG3518, COG3519, and COG3520), and finally the effectors *hcp* and *vgrG* (COG3157 COG3501) (Appendix Table A3).

Additionally, we found an unusual COG category (COG2885) in T6SS‐2 and T6SS‐3, adjacently to core genes. Besides the presence of conserved genes in the three clusters, the identity between them is very low (<50%) indicating that they may have different origins. Blastn analysis against GenBank nonredundant (nr) database shows that cluster T6SS‐1 shares high similarity and coverage (>70%) with a *P*. *phymatum* STM815^T^ plasmid, *Cupriavidus necator* H19, and *Burkholderia* sp. SCCGE1002. These bacterial species are found in soil and have the ability to nodulate in legumes and to accumulate polyhydroxybutyrate (PHB) under stress conditions (Ormeño‐Orrillo et al., 2012; Tee et al., 2017). On the other hand, the regions comprising T6SS‐2 and T6SS‐3 show a higher identity to *B. cenocepacia* and *P*. *glumae* T6SS. Finally, several *hcp* and *vgrG* orphan genes were also found, mapping outside these T6SS clusters, which might be functional.

## 4 CONCLUSIONS

Over the last two decades, *Paraburkholderia* gained considerable importance for their ability to fix nitrogen, promote plant growth and degrade recalcitrant chemical compounds. Approaches employing PGPB emerge as sustainable alternatives to chemical fertilizers in agriculture, and also bioremediation of impacted environments. However, many aspects of genetics, physiology, and biochemistry of beneficial plant‐bacteria endophytic interaction are not yet established. The genome sequencing and characterization of *P*. *kururiensis* type‐strain KP23^T^ allowed its confrontation with other available *P*. *kururiensis* genomes (M130 and ATSB13^T^) from different geographic regions. Comparative genomic analyses of these three genomes revealed important features. They share about 70% of their genome content, which includes genes involved in plant growth, such as ACC deaminase, genes for biosynthesis of IAA, multiple efflux pumps, dioxygenases, and genes involved in degradation of aromatic compounds. Interestingly, a wide repertoire of genes for protein secretion systems (T2SS, T3SS, T4SS, T5SS, and T6SS) was characterized. Some of these protein secretion systems may be involved in molecular cross‐talks with plant hosts and members of the surrounding microbiome. In summary, this study highlights the potential of *P. kururiensis* as a model to investigate adaptation mechanisms, and application in environmentally friendly strategies, such as bioremediation, biofertilization, and biocontrol of plant pathogens.

## CONFLICT OF INTERESTS

The authors declare no conflict of interest.

## AUTHORS CONTRIBUTION

GMD performed the bioinformatic analysis. ASP and MRC helped in the data analysis, VSG contributed to genome assembly and annotation of genomes analyzed. LFV performed molecular labwork. BCN and GMD conceived, designed the study and wrote the manuscript draft. All authors read and approved the final manuscript.

## ETHICS STATEMENT

None required.

## Data Availability

The genome sequence data of the type strain KP23^T^ has been deposited at DDBJ/EMBL/GenBank under the accession RJZE00000000. The version described in this paper is version RJZE01000000.

## APPENDIX 1

### 1.1

**Table A1:** Predicted genes involved to aromatic compounds in *P. kururiensis* genomes

gene name	Product	Genomes		
		KP23	M130	ATSB13^T^
*pcaR*	Pca regulon regulatory protein	x	x	x
*mhpA*	3‐(3‐hydroxy‐phenyl)propionate/3‐hydroxycinnamic acid hydroxylase	x	x	x
*mhpB*	2,3‐dihydroxyphenylpropionate/2,3‐dihydroxicinnamic acid 1,2‐dioxygenase	x	x	x
*mhpD*	2‐keto‐4‐pentenoate hydratase	x	x	x
*mhpF*	Acetaldehyde dehydrogenase	x	x	x
*mhpE*	4‐hydroxy‐2‐oxovalerate aldolase	x	x	x
*mhpC*	2‐hydroxy‐6‐oxononadienedioate/2‐hydroxy‐6‐oxononatrienedioate hydrolase	x	x	x
*hcaR*	Hca operon transcriptional activator HcaR	x	x	x
*hcaE*	3‐phenylpropionate/cinnamic acid dioxygenase subunit alpha	x	x	x
*hcaF*	3‐phenylpropionate/cinnamic acid dioxygenase subunit beta	x	x	x
*hcaC*	3‐phenylpropionate/cinnamic acid dioxygenase ferredoxin subunit	x	x	x
*hcaB*	3‐phenylpropionate‐dihydrodiol/cinnamic acid‐dihydrodiol dehydrogenase	x	x	x
*hcaD*	3‐phenylpropionate/cinnamic acid dioxygenase ferredoxin–NAD(+) reductase component	x	x	x
*‐*	Phenol hydroxylase P5 protein	x	x	x
*‐*	Phenol 2‐monooxygenase	x	x	x
*‐*	Toluene‐4‐monooxygenase system protein A	x	x	x
*‐*	Phenol hydroxylase P2 protein	x	x	x
*‐*	Phenol hydroxylase P1 protein	x	x	x
*boxA*	Benzoyl‐CoA oxygenase component A	x	x	x
*boxB*	Benzoyl‐CoA oxygenase component B	x	x	x
*boxC*	Benzoyl‐CoA‐dihydrodiol lyase	x	x	x
*boxD*	Shikimate kinase	x	x	x
*boxE*	3,4‐dehydroadipyl‐CoA semialdehyde dehydrogenase	x	x	x
*‐*	Benzoate–CoA ligase	x	x	x
*‐*	2‐succinyl‐6‐hydroxy‐2,4‐cyclohexadiene‐1‐carboxylate synthase	x	x	x
*tmoA*	Toluene‐4‐monooxygenase system protein A	x	x	x
*tmoB*	Toluene‐4‐monooxygenase system protein B	x	x	x
*tmoC*	Toluene‐4‐monooxygenase system ferredoxin subunit	x	x	x
*tmoD*	Toluene‐4‐monooxygenase system protein D	x	x	x
*tmoE*	Toluene‐4‐monooxygenase system protein E	x	x	x
*tmoF*	Toluene‐4‐monooxygenase, subunit TmoF	x	x	x
*‐*	2,3‐dihydroxyphenylpropionate/2,3‐dihydroxicinnamic acid 1,2‐dioxygenase	x	x	x
*‐*	4‐hydroxy‐4‐methyl‐2‐oxoglutarate aldolase/4‐carboxy‐4‐hydroxy‐2‐oxoadipate aldolase	x	x	x
*‐*	4‐hydroxy‐4‐methyl‐2‐oxoglutarate aldolase/4‐carboxy‐4‐hydroxy‐2‐oxoadipate aldolase	x	x	x
*‐*	4‐hydroxy‐4‐methyl‐2‐oxoglutarate aldolase/4‐carboxy‐4‐hydroxy‐2‐oxoadipate aldolase	x	x	x
*‐*	Phthalate 4,5‐dioxygenase oxygenase subunit	x	x	x
*‐*	4‐hydroxybenzoate 3‐monooxygenase (NAD(P)H)	x	x	x
*‐*	3‐oxoadipate CoA‐transferase subunit A	x	x	x
*‐*	3‐oxoadipate CoA‐transferase subunit B	x	x	x
*‐*	3‐carboxy‐cis,cis‐muconate cycloisomerase	x	x	x
*‐*	3‐oxoadipate enol‐lactonase 2	x	x	x
*hbzD*	3‐hydroxybenzoate 6‐hydroxylase 1	x	x	x
*hbzE*	Gentisate 1,2‐dioxygenase	x	x	x

**Table A2:** Distribution of Type Secretion Systems on *Paraburkholderia kururiensis* genomes

Genes	*Paraburkholderia kururiensis* strains		
	KP23	M130	ATSB13^T^
**T2SS**			
T2SS_gspN	x	x	‐
T2SS_gspM	x (2)*	x (2)*	x
T2SS_gspL	x (2)*	x	x
T2SS_gspK	x	x	‐
T2SS_gspJ	x (2)*	x (2)*	x
T2SS_gspI	x (2)*	x (2)*	x
T2SS_gspH	x(2)*	x (2)*	x
T2SS_gspG	x (2)*	x (2)*	x
T2SS_gspC	x (2)*	x (2)*	x
T2SS_gspF	x (2)*	x (2)*	x
T2SS_gspE	x	x	‐
T2SS_gspD	x (2)*	x (2)*	x
hypothetical protein	10 | 16*	15 | 11*	15
**T3SS**			
T3SS_sctC	x	x	x
T3SS_sctT	x	x	x
T3SS_sctN	x	x	x
T3SS_sctJ	x	x	x
T3SS_sctU	x	x	x
T3SS_sctV	x	x	x
T3SS_sctR	x	x	x
T3SS_sctS	x	x	x
T3SS_sctQ	‐	x	x
hypothetical protein	19	18	20
**T4SS 2**			
T4SS_virB3	x	‐	‐
T4SS_cagE/trbE/virB	x	‐	‐
T4SS_trbL/virB6	x	‐	‐
T4SS_virB11/trbB	x	‐	‐
hypothetical protein	2	‐	‐
**T5SS**			
T5aSS			
T5aSS_PF03797	x (2)*	x (2)*	‐
hypothetical protein	10 | 10*	10 | 10*	‐
T5bSS			
T5bSS_translocator	x (8)*	x (3)*	x (3)*
hypothetical protein	10 (8)*	10 (3)*	10 (3)*
T5cSS			
T5cSS_PF03895	x (5)*	x (3)*	x (4) *
hypothetical protein	10 (5)*	10 (3)*	10 (4)*
**T6SS**			
T6SSi			
T6SSi_tssM	x (2)*	x	x
T6SSi_tssL	x	x	x
T6SSi_tssH	x (2)*	x	x
T6SSi_tssG	x (2)*	x	x
T6SSi_tssF	x (2)*	x	x
T6SSi_tssE	x (2)*	‐	x
T6SSi_tssD	x	x	x
T6SSi_tssC	x	x	x
T6SSi_tssB	x	x	x
T6SSi_tssJ	x (2)*	x	x
T6SSi_tssK	x (2)*	x	x
T6SSi_tssL*	x (2)*	x	x
T6SSi_tssA	x	‐	‐
T6SSi_evpJ	x	‐	‐
hypothetical protein	13 | 15*	13	13

**Table A3:** Identification functional of T6SS core genes according COG categories

COG categories	gene name	T6SS‐1	T6SS‐2	T6SS‐3
COG3515	*bimE*	x	x	x
COG3523	*tssM*	x	x	x*
COG3455	*tssL*	x	x	x
COG3522	t*ssK*	x	x	x
COG3501	*vgrG*	x	‐	x*
COG3520	*tssE*	x	x	x*
COG3519	*tssD*	x	x*	x*
COG3518	*tssC*	x	x	x*
COG3517	*tssB*	x	x	x*
COG3516	*tssA*	x	x	x*
COG3157	*hcp*	x	x	x
COG0542	*clpV*	x	x	x*
COG3913	*impP*	‐	x	‐
COG2885	*ompA*	‐	‐	x

* Genes present differences in the size or in the order of cluster – absence of genes
